# Environmental Remediation Potential of Ferrous Sulfate Waste as an Eco-Friendly Coagulant for the Removal of NH_3_-N and COD from the Rubber Processing Effluent

**DOI:** 10.3390/ijerph182312427

**Published:** 2021-11-25

**Authors:** Muhammad Khalish Mohammad Ilias, Md. Sohrab Hossain, Rahmat Ngteni, Adel Al-Gheethi, Harlina Ahmad, Fatehah Mohd Omar, Mu. Naushad, Sadanand Pandey

**Affiliations:** 1 Environmental Technology Division, School of Industrial Technology, Universiti Sains Malaysia, Gelugor 11800, Penang, Malaysia; muhdkhalish.ilias@student.usm.my (M.K.M.I.); harlinaa@usm.my (H.A.); 2 Sime Darby Research Sdn Bhd, Carey Island 42900, Selangor, Malaysia; rahmat.ngteni@simedarbyplantation.com; 3 Micro-Pollutant Research Centre (MPRC), Department of Civil Engineering, Faculty of Civil Engineering and Built & Environment, Universiti Tun Hussein Onn Malaysia, Parit Raja 86400, Johor, Malaysia; adel@uthm.edu.my; 4 School of Civil Engineering, Universiti Sains Malaysia, Nibong Tebal 14300, Penang, Malaysia; cefatehah@usm.my; 5 Department of Chemistry, College of Science, King Saud University, P.O. Box 2455, Riyadh 11451, Saudi Arabia; mnaushad@ksu.edu.sa; 6 Department of Chemistry, College of Natural Science, Yeungnam University, Gyeongsan 38541, Korea; sadanand.au@gmail.com

**Keywords:** industrial effluent, rubber processing effluent, coagulation, FeSO_4_·7H_2_O waste, eco-friendly coagulant

## Abstract

The present study was conducted to determine the potential of utilizing the FeSO_4_·7H_2_O waste from the titanium manufacturing industry as an effective coagulant for treating industrial effluent. In this study, the secondary rubber processing effluent (SRPE) was treated using ferrous sulfate (FeSO_4_·7H_2_O) waste from the titanium oxide manufacturing industry. The FeSO_4_·7H_2_O waste coagulation efficiency was evaluated on the elimination of ammoniacal nitrogen (NH_3_-N) and chemical oxygen demand (COD) from SRPE. The central composite design (CCD) of experiments was employed to design the coagulation experiments with varying coagulation time, coagulant doses, and temperature. The coagulation experiments were optimized on the optimal elimination of NH_3_-N and COD using response surface methodology (RSM). Results showed that coagulant doses and temperature significantly influenced NH_3_-N and COD elimination from SRPE. The highest NH_3_-N and COD removal obtained were 98.19% and 93.86%, respectively, at the optimized coagulation experimental conditions of coagulation time 70 min, coagulant doses 900 mg/L, and temperature 62 °C. The residual NH_3_-N and COD in treated SPRE were found below the specified industrial effluent discharge limits set by DoE, Malaysia. Additionally, the sludge generated after coagulation of SRPE contains essential plant nutrients. The present study’s finding showed that FeSO_4_·7H_2_O waste generated as an industrial byproduct in a titanium oxide manufacturing industry could be utilized as an eco-friendly coagulant in treating industrial effluent.

## 1. Introduction

The increasing production of rubber products leads to the rapid growth of rubber industries to meet economic demands. Rubber processing requires a vast quantity of freshwater, and therefore the rubber processing industries are generating a massive amount of industrial effluent [1,2]. It is estimated to generate about 20,500 L of rubber effluents during processing 1 tonne of rubber [2]. The rubber effluents contain a high concentration of various inorganic and organic contaminants. Therefore, the discharge of untreated rubber effluent into a watercourse would raise serious environmental pollution concerns [3]. Hence, there is an urgency to determine an effective treatment process for the safe discharge of rubber processing effluent to eliminate undesirable substances to preserve the aquatic organisms.

The wastewater treatment mainly allows discharging industrial effluents without posing detrimental effects to the natural environment and human health since the effluent is discharged to a watercourse. However, the disposal of treated or partially treated industrial effluent in a watercourse can affect the aquatic ecosystem by depleting dissolved oxygen in the water [3,4]. Malaysian rubber processing industry implements biological wastewater treatment methods, including aerobic and anaerobic digestion, to treat the rubber processing effluent [4,5]. However, the biological wastewater treatment methods for industrial effluent treatment have several limitations, including the requirements of large food prints and long hydraulic retention time [4,6]. The biological treatment process of industrial effluent cannot minimize the concentration of the residual pollutants below the recommended discharge limits suggested by the Department of Environment (DoE), Malaysia (DoE 2009). Therefore, the biologically treated industrial effluent requires further treatment before discharging in a watercourse to diminish the pollutant concentrations lower the specified discharge limits set by DoE, Malaysia. 

Coagulation–flocculation is the utmost effective technique to eliminate the organic and inorganic contaminants from wastewater [7,8]. However, the coagulation of industrial effluents is generally carried out by commercially available coagulants, including iron salts, alum, aluminum chloride, and polymer-based coagulants [2,8,9]. However, using these commercial coagulants in industrial effluent treatment is a costly process due to the high price of commercial metals and polymeric-based coagulants [9]. Furthermore, the aluminum-based coagulant application generates hazardous sludge, which requires costly disposal [8,9]. Therefore, scientists and environmentalists are searching for an alternative to commercial coagulants for treating industrial effluents. However, there is an increasing interest in utilizing waste materials to enhance sustainability and minimize environmental pollution [7,10]. FeSO_4_·7H_2_O is an industrial byproduct of the titanium oxide manufacturing industry, generated during extracting the titanium pigment from ilmenite ore in the sulfate method [7,11]. The titanium oxide industries are generating a vast amount of FeSO_4_·7H_2_O waste as a byproduct and storing it at the industry premises because of its high disposal cost. Thus, there is a need for an effective and environmentally friendly disposal method or the sustainable utilization of this massive volume of FeSO_4_·7H_2_O waste [11]. 

Several approaches have been implemented to enhance the sustainable utilization of FeSO_4_·7H_2_O waste, such as chemical reductant [12], raw materials for polymeric sulfate synthesis [13], and sodium–ferrate synthesis [14]. All these approaches are not sufficient to meet up the challenges of managing the massive volume of FeSO_4_·7H_2_O waste in the titanium oxide manufacturing industry, urging further implementation of FeSO_4_·7H_2_O waste to enhance sustainability [7,11]. However, FeSO_4_·7H_2_O waste has the comparable coagulation efficiency of aluminum-based coagulants, and the sludge generated after coagulation is not considered a hazardous element, especially since iron is the essential micronutrient in plant growth and fruits production [15]. Thus, implementing the FeSO_4_·7H_2_O wastes as an eco-friendly coagulant in SRPE treatment bears a considerable interest in enhancing sustainable utilization of industrial byproducts and minimizing the hazardous sludge generation from the industrial treatment. 

Many parameters, such as coagulant doses, treatment time, temperature, and pH, play an important role in enhancing or maximizing coagulation efficiency. Thus, the FeSO_4_·7H_2_O waste coagulation process variables urge a quantitative assessment to improve the NH_3_-N and COD removal from SRPE. Further, the evaluation of coagulation appliances is a convenient tool to determine the suitability and coagulant potential for a coagulation process [16]. The conventional experimental method implies that one parameter at a time could avoid some critical features of the experiments, such as the quadratic effect of the parameters and the interaction effect between or among the parameters [16,17]. Response surface methodology (RSM) is a widely applied statistical and mathematical method for analyzing and modeling a process behavior to assess the influences of several parameters and simultaneously optimize their responses [18]. In a recent study, Ngteni et al. [2] employed the FeSO_4_·7H_2_O waste to eliminate biological oxygen demand(BOD), chemical oxygen demand (COD), suspended solids (SS), and NH_3_-N from SPRE with varying pH, coagulant doses, treatment time, and temperature. It was found that pH, coagulant doses, temperature, treatment time, and sedimentation time have potentially influenced the elimination of NH_3_-N and COD from SPRE. The NH_3_-N and COD removal increased with increasing coagulant doses, temperature, and treatment time. However, the NH_3_-N and COD removal increased with increasing pH up to pH 5.0, and the removal was negligible over 60 min of sedimentation time. Although various parameters influenced the FeSO_4_·7H_2_O coagulation efficiency, the residual NH_3_-N and COD were not reduced below the suggested discharge limits for the residual NH_3_-N and COD concentration in treated rubber effluent set by DoE, Malaysia. 

The aim of this study is to promote the sustainable utilization of FeSO_4_·7H_2_O waste from the titanium manufacturing industry as an environmental remediation potential coagulant for wastewater treatment and to zero waste generation from industrial effluent treatment. Therefore, in the present study, the FeSO_4_·7H_2_O waste, obtained as a byproduct of the titanium oxide manufacturing industry, was employed as a coagulant to eliminate NH_3_-N and COD from the secondary rubber processing effluent (SRPE). The coagulation process was optimized using RSM based on the maximum elimination of NH_3_-N and COD from SRPE. Furthermore, elemental compositions and mineral compositions in generated sludge were determined using scanning electron microscopy with energy dispersive X-Ray Analysis (SEM-EDX), and inductively coupled plasma optical emission spectrometry (ICP-OES) was employed to determine the possible utilization of the generated sludge as an organic fertilizer. The findings of the present study will be helpful in determining alternatives to commercial inorganic coagulants in treating industrial effluent without generating toxic sludge.

## 2. Materials and Methods

### 2.1. Sample Collection 

SRPE was collected from Sime Darby Latex Sdn.Bhd-Batu Anam, Segamat, Johor, Malaysia. The physicochemical properties of the collected SPRE are shown in Table 1. The collected SRPE was stored at 4 °C prior to conducting experiments. The FeSO_4_·H_2_O waste was obtained from the Venator Asia Sdn Bhd, Terengganu, Malaysia. The typical analyses of the FeSO_4_·7H_2_O waste are discussed elsewhere, wherein the percentage Fe(II) content was determined to be 18.05 ± 0.78%.

### 2.2. Determination of NH_3_-N and COD Concentrations

The NH_3_-N and COD concentrations in untreated and treated SRPE were determined following the method described by Ngteni et al. [2]. COD was determined using the reactor digestion method (HACH method, 8000) with a HACH DR 2800 spectrometer (HACH company, Loveland, CO, USA) equipped with high range (HR) COD digestion vials (range 200–15,000 mg/L). Subsequently, 2 mL of homogenized sample and deionized water were added to HR COD digestion vials for samples and blank tests. The vials were then placed in a preheated COD reactor (DRB 200, HACH company, Loveland, CO, USA) and heated to 150 °C for 2 h. The vials were then cooled at room temperature, and COD (mg/L) was determined using a DR 2800. The NH_3_-N content of treated and untreated SRPE was determined using the salicylate technique using ammonia salicylate and ammonia cyanurate reagents powder pillows. The data logging and measurement of NH_3_-N (mg/L) were performed using an advanced water quality laboratory spectrophotometer (HACH-DR 900, HACH company, Loveland, CO, USA). Prior to analysis, the pH of treated and untreated SRPE was warmed and neutralized with a concentrated (5 M) sodium hydroxide solution. The spectrometer’s wavelength was set to 655 nm, and the cell compartment was filled with a 10 mL cell riser. The sample cells were then filled with 0.1 mL of sample and 10 mL of blank sample (distilled water was used as a control). A pillow of ammonia salicylate reagent powder was added to the sample and agitated for 3 min. Later, the same sample cell was agitated for 15 min with ammonia cyanurate reagent powder. After zeroing the spectrophotometer with a blank sample, the sample concentration of NH_3_-N was determined in mg/L.

### 2.3. Coagulation Experiments Procedure 

The experiments were carried out using a fabricated jar test apparatus varying flocculation time (30–60 min), coagulant doses (0.5–1.0 g/L), and temperature (40–60 °C). The constant parameters were pH (pH 5.0), the rapid mixing time of 3 min at 200 rpm, and the settling time of 60 min. The percentage elimination of NH_3_-N and COD was determined using the Equation (1):

(1) $$\mathrm{Removal}\;(\%)\;=\left(1-\frac{C_{t}}{C_{i}}\right)\times 100$$

where C_i_ initial NH_3_-N and COD concentrations in untreated SRPE, and C*_t_* is final NH_3_-N and COD concentrations in treated SRPE. 

### 2.4. Optimization Using Response Surface Methods

The coagulation experiments were designed following CCD for three factors and two variables, and the coagulation experimental conditions were optimized using RSM. The statistical analysis of the influence of the parameters for eliminating NH_3_-N and COD from SRPE was evaluated using the second-order quadratic model. The parameters such as coagulation time, coagulant doses, and temperature were independent, and these parameters were coded using Equation (2):

(2) X=[xmax+xmin]2[xmax−xmin]2

where *x* and *X* are natural variables and coded variables, respectively; *x*_min_ is a low level of the variables, and *x*_max_ is the high level of the variables. Table 2 shows the high, intermediate, and low levels of the parameters were labeled as +1, 0, and −1, respectively. The FeSO_4_·7H_2_O waste coagulation efficiency on NH_3_-N and COD removal from SRPE was described using the second-order polynomial equation, as shown in Equation (3):

(3) $$Y=\beta_{0}+\sum_{i=1}^{n}\beta_{i}X_{i}+\sum_{i≺1}^{n}\beta_{ij}X_{ij}+\sum_{i=1}^{n}\beta_{ii}X_{i}^{2}$$

where *Y* is the predicted response for NH_3_-N and COD removal from SPRE, *β*_0_ refers to the constant coefficient of intercept, *β_i_* denotes the linear terms of the variables, *β_ij_* denotes the interaction terms of the variables, and *β_ii_* denotes the quadratic terms of the second-order polynomial equation. The experimental data were analyzed and fitted with the second-order polynomial equation using Design Expert Software (ver.11) (Stat-Ease, Inc., Minneapolis, MN, USA). The effect of the three parameters and their significant effect on the elimination of NH_3_-N and COD were examined using the analysis of variance (ANOVA) at a 95% of confidence level (*p* < 0.05). The determination coefficient (*R*^2^) and adjusted determination coefficient (*R*^2^*adj*) were used to assess the accuracy of fitting the regression model with the experimental data. The synergistic effects of the coagulation parameters and the interaction effects between the parameters were evaluated using the second-order polynomial regression model. The interaction effect between or among independent variables was described using response surface plots on NH_3_-N and COD removal from SPRE.

### 2.5. Sludge Characterization

The surface morphology of FeSO_4_·7H_2_O waste and the sludge generated after coagulation were determined using field-emission scanning electron microscopy (FE-SEM) (Quanta FEG 650, FEI, Hillsboro, OR, USA). The sample was taken on an aluminum stub, then mounted with double side carbon tape, and sputter-coated with gold powder using a high-resolution sputter coater. The SEM image was taken at 10,000 magnification and an accelerating voltage of 5 kV. Moreover, the elemental compositions in FeSO_4_·7H_2_O waste and the sludge generated after coagulation were determined by Energy Dispersive X-Ray (EDX) analyses (Quanta FEG 650, FEI, Hillsboro, OR, USA). The presence of minerals compositions such as boron (b), calcium (Ca), iron (Fe), magnesium (mg), phosphorus (P), and Potassium (K) was identified by Inductively Coupled Plasma, equipped with the Optical Emission Spectroscopy (ICP-OES) (PerkinElmer Optima 7300 DV, Waltham, MA, USA).

## 3. Results and Discussions 

### 3.1. Removal of NH_3_-N and COD from SPRE

Since the coagulation efficiency depends on the variation of the parameters, optimizing the FeSO_4_·7H_2_O waste coagulation experimental conditions is an essential step in maximizing the percentage NH_3_-N and COD elimination from SPRE. The experimental conditions of FeSO_4_·7H_2_O waste coagulation were optimized by assessing the influence of the studied independent variables such coagulation time (min), coagulant doses (mg/L), and temperature (°C) on the percentage NH_3_-N and COD elimination from SPRE. During coagulation experiments, the sedimentation time (60 min) and pH (pH 5.0) parameters were kept constant. The coagulation experiments were designed using the central composite design (CCD) based on the coded values of three independent parameters and have resulted in 20 experimental runs. Table 3 shows the actual and predicted values of 20 experimental runs based on CCD on removing NH_3_-N and COD from SRPE using FeSO_4_·7H_2_O waste as a coagulant. 

It was observed that the studied independent parameters have expressively influenced the percentage elimination of NH_3_-N and COD from SRPE, suggesting they could be further enhanced to minimize the residual NH_3_-N and COD concentration in treated SRPE below the discharge limits suggested by DOE, Malaysia. Moreover, the actual experimental values and predicted values were in good agreement, and the highest percentage of NH_3_-N and COD removal from SRPE obtained was 95.10% and 91.83%, respectively, at high levels of coagulation time, coagulant doses, and temperature. The findings in Table 3 indicate the suitability and aptness of the proposed second-order polynomial equation for modeling and analyzing the influence of the process parameters towards the responses [17,18].

### 3.2. Regression Model for Response 

A regression equation was simulated from the experimental data designed using CCD following the second-order quadratic equation. Furthermore, the regression equation was derived at the end of the coagulation experiment using the quadratic, interaction, and linear function of the CCD of the experimental data for eliminating NH_3_-N and COD and from SPRE. The aptness and signal-to-noise ratio of the regression equation for the response prediction were evaluated using F-value. For instance, a low value of the standard error and a high coefficient of F-value indicate minimal noise in the regression model equation, implying the suitability and soundness of the model for the prediction of the response [19]. Table 4 shows that the values of coefficient, standard error, F-value, and *p*-value. These indicator values were utilized to evaluate the validity of the prediction of the model for the percentage elimination of NH_3_-N and COD from SRPE using FeSO_4_·7H_2_O waste as a coagulant. 

The coagulation efficiency of FeSO_4_·7H_2_O for the percentage elimination of NH_3_-N and COD from SRPE was evaluated with the regression coefficients analysis of the linear, quadratic, and interaction effects of the parameters. It was found that the linear terms such as coagulation time, coagulant doses, and temperature, as well as the interaction effects between temperature and coagulation time, and the quadratic terms of temperature and coagulant doses, were statistically significant on the COD removal from SRPE. In the case of NH_3_-N removal from SRPE, the linear terms of temperature and coagulant doses, the interaction effects between coagulant doses and coagulation time, and the quadratic terms of temperature and coagulant doses were statistically significant.

The significant negative quadratic interaction coefficient between temperature and coagulant doses inhibited the FeSO_4_·7H_2_O waste coagulation. However, the negative coefficient of the interaction between the temperature and coagulation time was insignificant, indicating that the increased coagulation time at a higher temperature did not influence the percentage NH_3_-N and COD elimination from SRPE. The statistically significant second-order quadratic model reveals the aptness of the variables for the elimination of NH_3_-N and COD from SRPE. The second-order quadratic model can be represented in terms of coded factors, as shown in Equations (4) and (5):

(4) $$\begin{array}{ll} Y_{COD} & =87.97+1.77x_{1}+3.71x_{2}+1.84x_{3}-0.27x_{1}^{2}-3.78x_{2}^{2}-0.70x_{3}^{2}+0.041x_{1}x_{2}\\ & -0.51x_{1}x_{3}+0.47x_{2}x_{3} \end{array}$$

(5) $$\begin{array}{ll} Y_{NH_{3}-N} & =89.49+0.92x_{1}+4.46x_{2}+3.96x_{3}-0.47x_{1}^{2}-6.90x_{2}^{2}-2.57x_{3}^{2}+7.06x_{1}x_{2}\\ & -1.54x_{1}x_{3}+0.43x_{2}x_{3} \end{array}$$

where *Y_COD_* and *Y_NH3-N_* refer to yield for the percentage elimination of the COD and NH_3_-N from SPRE. Table 5 shows the analysis of variance (ANOVA) analyses for the NH_3_-N and COD removal from SRPE. The ANOVA analyses indicate that the predicted regression model for eliminating NH_3_-N and COD from SRPE was significant at a 95% confidence level (*p* < 0.05). The determination coefficient (*R*^2^) values for the NH_3_-N and COD removal from SRPE were 0.9047 and 0.9756, respectively. The determination of adjusted coefficients (*R*^2^*_adj_*) was found to be 0.8190 and 0.9536 for the NH_3_-N and COD removal, respectively. The obtained *R*^2^ and *R*^2^*_adj_* values for the NH_3_-N and COD removal from SRPE using FeSO_4_·7H_2_O waste as a coagulant showed the high correlation between the experimental and predicted values, which indicates the suitability and aptness of the second-order polynomial regression model for analyses and the prediction. The insignificant lack of fit implies that the prediction conducted by the regression model was accurate within the levels of the parameters investigated. It was found that the effects of the interactions between coagulation parameters were synergistic, implying that the accumulation of the influence of studied parameters would enhance the FeSO_4_·7H_2_O waste coagulation efficiency. 

### 3.3. Response Surface Analyses

**Figure 1:**
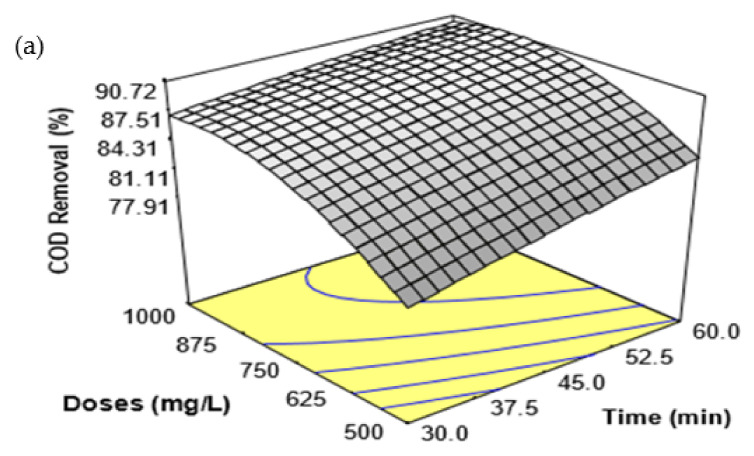

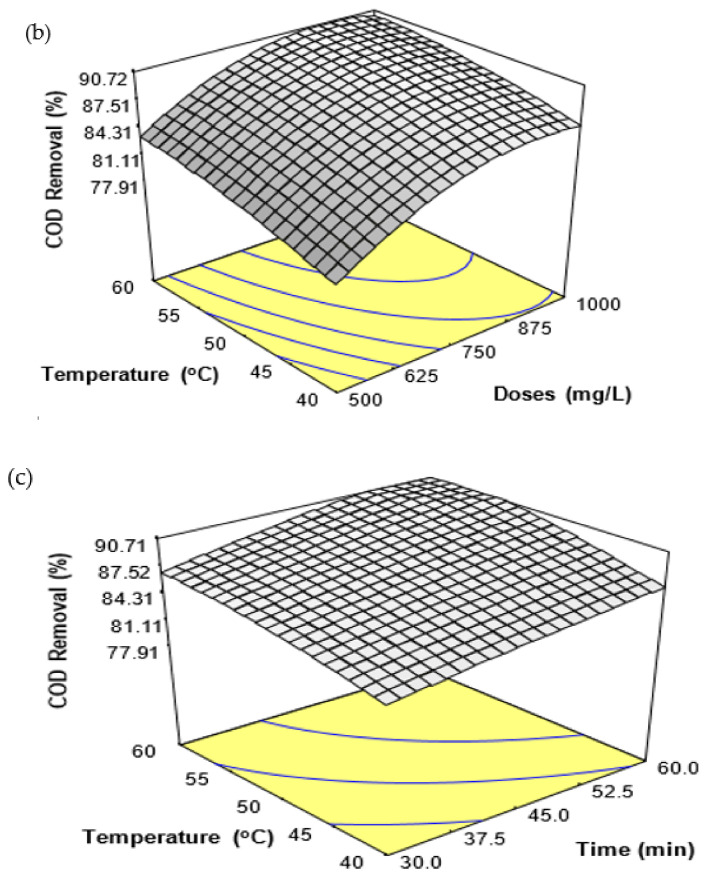
Response surface plots for the removal of COD from SRPE using FeSO_4_·7H_2_O waste as a coagulant. (**a**) Interaction effect between coagulation time and FeSO_4_·7H_2_O waste doses, (**b**) Interaction effect between FeSO_4_·7H_2_O waste doses and temperature, and (**c**) Interaction between coagulation time and temperature.

Figure 1 shows the response surface plot for the interaction effects between coagulation time and coagulant doses (Figure 1a), coagulant doses and temperature (Figure 1b), and coagulation time and temperature (Figure 1c) for the percentage elimination of COD from SRPE. It was observed that the interaction effect between coagulant time and doses (Figure 1a) was statistically significant for the removal of COD from SRPE. The interaction effect between FeSO_4_·7H_2_O waste doses and temperature (Figure 1b) and between coagulation time and temperature (Figure 1c) were insignificant for the elimination of COD from SRPE. The percentage elimination of COD increased by increasing the coagulant doses at a higher coagulation time (Figure 1a). However, the percentage removal of COD was negligible with an increase of coagulation time at higher coagulant doses. The interaction effects between coagulant doses and temperature (Figure 1b) reveal that the percentage of COD removal from SRPE increased with an increasing FeSO_4_·7H_2_O waste dose at a higher temperature up to 900 mg/L coagulant doses; subsequently, the percentage of COD removal was negligible with further increase of coagulant doses. However, the percentage of COD removal was increased with increasing temperature at higher coagulant doses. The highest COD removal obtained was about 91% at coagulant doses of 0.9 g/L, at a temperature of 60 °C, for the 45 min of coagulation time. However, the percentage of COD removal increased slightly with an increase in temperature at a longer treatment time (Figure 1c). At a higher temperature, the percentage of COD removal was negligible with an increase in coagulation time. The uppermost COD removal (over 90%) was achieved at coagulation time 60 min, coagulant doses 0.75 g/L, and at a temperature of 60 °C. 

**Figure 2:**
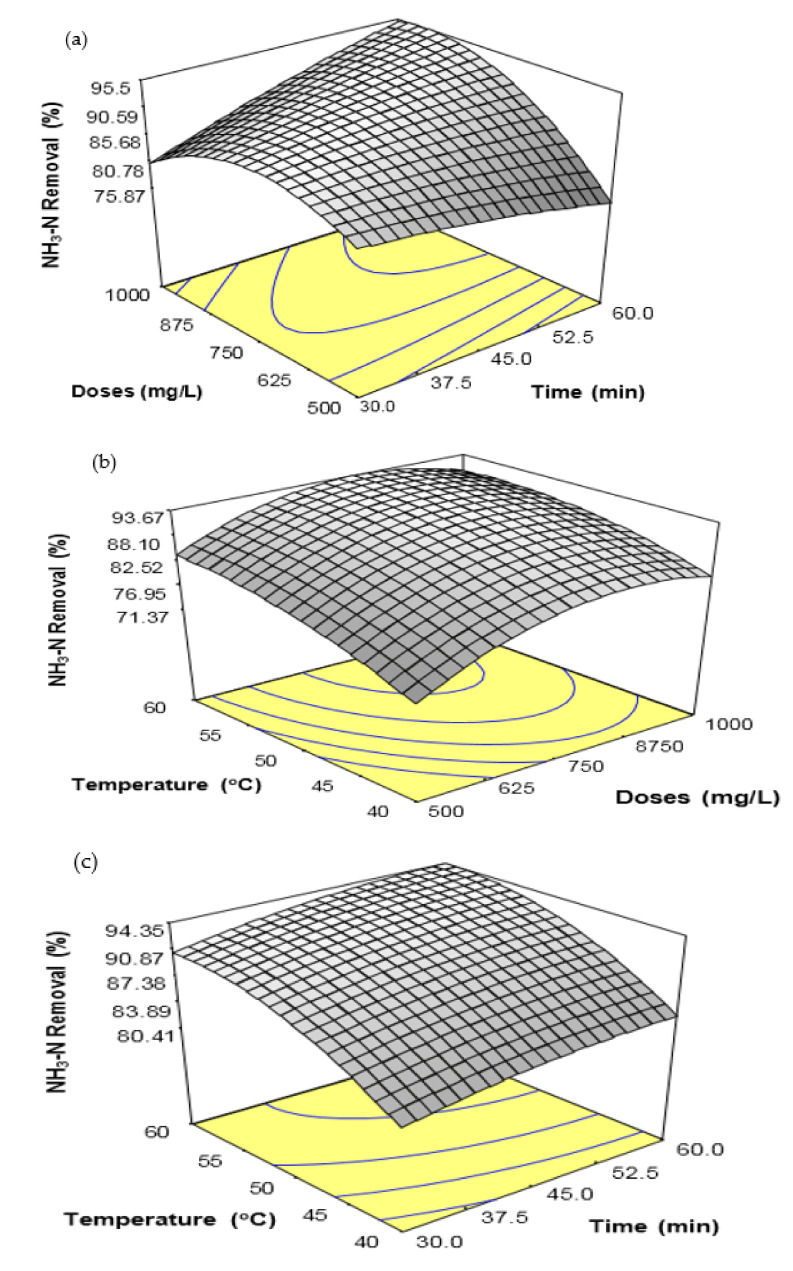
Response surface plots for the removal of NH_3_-N from SRPE using FeSO_4_·7H_2_O waste as a coagulant. (**a**) Interaction effect between coagulation time and FeSO_4_·7H_2_O waste doses, (**b**) Interaction effect between FeSO_4_·7H_2_O waste doses and temperature, and (**c**) Interaction between coagulation time and temperature.

Figure 2 shows the response surface plot for the interaction effects between coagulation time and coagulant doses (Figure 2a), coagulant doses and temperature (Figure 2b), and coagulation time and temperature (Figure 2c). The interaction effect between coagulant doses and coagulation time was statistically significant on the percentage of NH_3_-N removal from SRPE (Figure 2a). However, the interaction effects between coagulant doses and temperature (Figure 2b) and between coagulation time and temperature (Figure 2c) were statistically insignificant for the percentage NH_3_-N removal from SRPE. At high coagulation time, the percentage of NH_3_-N removal was increased with increasing coagulant doses up to 875 mg/L (Figure 2a) and decreased after that. The highest percentage of NH_3_-N removal from SRPE obtained was over 95% at coagulant doses of 875 mg/L, coagulation time of 60 min, and at a temperature of 45 °C. However, the percentage of NH_3_-N removal from SRPE was not influenced by the increasing coagulation time at a higher coagulant dose. The interaction effect between coagulant doses and temperature on the percentage of NH_3_-N removal from SRPE (Figure 2b) shows that the increase in temperature slightly increased the NH_3_-N removal at a higher coagulant dose. 

Conversely, the percentage of NH_3_-N removal from SRPE was increased with increasing coagulant doses up to 750 mg/L coagulant doses at a higher temperature. The highest increase of about 93% of NH_3_-N removal was obtained at coagulant doses of 875 mg/L, temperature of 60 °C, and coagulation time of 45 min. Figure 2c presents the interaction effect between coagulation time and temperature for the percentage of NH_3_-N removal from SRPE. It was found that the percentage of NH_3_-N removal from SRPE increased with increasing temperature from 40 °C to 55 °C at a higher coagulation time. However, the percentage of NH_3_-N removal was negligible with increasing coagulation time at a higher temperature. A maximum of about 95% of NH_3_-N elimination was obtained at coagulation time of 60 min, temperature of 60 °C, and coagulant doses of 750 mg/L. 

It was found that both temperature and coagulant doses played a significant role in eliminating NH_3_-N and COD from SRPE treated with FeSO_4_·7H_2_O waste as a coagulant. The removal of NH_3_-N and COD from SRPE with increasing temperatures can be attributed to the reduction of the viscosity of SRPE, which substantially increased the diffusion rate of the adsorbate to the boundary line of the coagulant (FeSO_4_·7H_2_O waste) [7,20,21]. Moreover, the increased kinetic energy of Fe(II) particles with a higher temperature facilitates collisions between the coagulant particles and suspended particles present in SRPE, which increases the NH_3_-N and COD removal. The increased FeSO_4_·7H_2_O waste coagulation efficiency with increasing doses heightens the Fe(II) concentration in the solution, which substantially increased the neutralization of negative charged organic particles [7,22,23] and ion exchange with positively charged inorganic particles for COD and NH_3_-N elimination from SRPE, respectively [24]. However, the decrease of the coagulation efficiency of FeSO_4_·7H_2_O waste with the increasing coagulant doses over 900 mg/L can be attributed to the increase of zeta potential of the surface of suspended and colloidal particles [25]. However, Mageshkumar and Karthikeyan [26] observed that the formation of the Brownian motion of the suspended organic particles present in the effluent with an excessive amount of the coagulant doses sustainably increases the kinetic energy on the surface of the coagulant and hence decreases the removal of organic particles from the effluent.

### 3.4. Optimization of FeSO_4_·7H_2_O Coagulation Process

Various parameters significantly influence the FeSO_4_·7H_2_O waste coagulation efficiency, thus optimizing the coagulation efficiency on the NH_3_-N and COD elimination from SRPE. In the present study, FeSO_4_·7H_2_O waste coagulation parameters such as coagulation time, coagulant doses, and temperature significantly influenced the COD removal from SRPE, while coagulant doses and temperature were significantly influential in removing NH_3_-N from SRPE. The Design Expert Software (Version 11) (Stat-Ease, Inc., Minneapolis, MN, USA) was used to optimize the FeSO_4_·7H_2_O coagulation parameters for the maximum removal of NH_3_-N and COD from SRPE, as shown in Table 6. The optimal experimental conditions were determined to be coagulation time 70 min, doses 900 mg/L, and temperature 62 °C. Under these experimental conditions, the removal of COD and NH_3_-N obtained were 93.86% and 98.19%, respectively. Rahman et al. [4] obtained a maximum of 87% COD removal from rubber processing effluent using a multiphase treatment scheme, such as upflow anaerobic sludge blanket treatment, flowed by coagulation and aeration. Ize-Iyamu et al. [27] obtained about 70% of COD removal from rubber processing effluent using chitosan as a biocoagulant. Thus, the higher COD removal obtained from rubber processing effluent using FeSO_4_·7H_2_O reveals that the FeSO_4_·7H_2_O can be used as an alternative to the commercial coagulant for effectively treating rubber processing effluent. 

In our previous study, the influence of BOD, COD, SS, and NH_3_-N removal from SRPE was determined with varying pH, FeSO_4_·7H_2_O doses, temperature, and treatment time [2]. The highest BOD, COD, SS, and NH_3_-N removal obtained were about 97%, 99%, 98%, and 95%, respectively, at pH 5.0, coagulant doses of 1 g/L, the temperature of 70 °C, and the coagulation time of 60 min. In the present study, it was found that higher NH_3_-N removal might be due to the extended coagulation time. Although temperature plays a significant role in FeSO_4_·7H_2_O waste coagulation for NH_3_-N and COD removal from SRPE, most of the studies in the literature have not considered the evaluation of the effect of temperature in the coagulation–flocculation process. The addition of temperature in the coagulation–flocculation process increases the treatment cost of the industrial effluent treatment due to energy consumption. However, the NH_3_-N removal from the industrial effluent is challenging [28]. Therefore, the inclusion of temperature in the coagulation–flocculation process must be considered to minimize the residual NH_3_-N in treated industrial effluent below the discharge limits set by respective environmental agencies. 

### 3.5. Evaluation of the FeSO_4_·7H_2_O Waste as an Eco-Friendly Coagulant 

**Figure 3:**
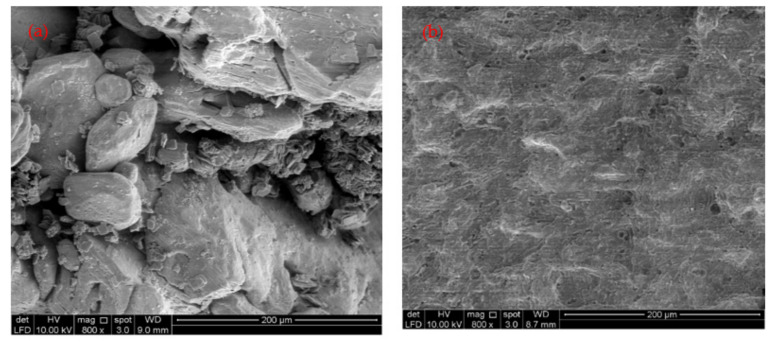
(**a**) SEM image of FeSO_4_·7H_2_O waste, and (**b**) generated sludge after SPRE treatment at optimized experimental conditions.

**Figure 4:**
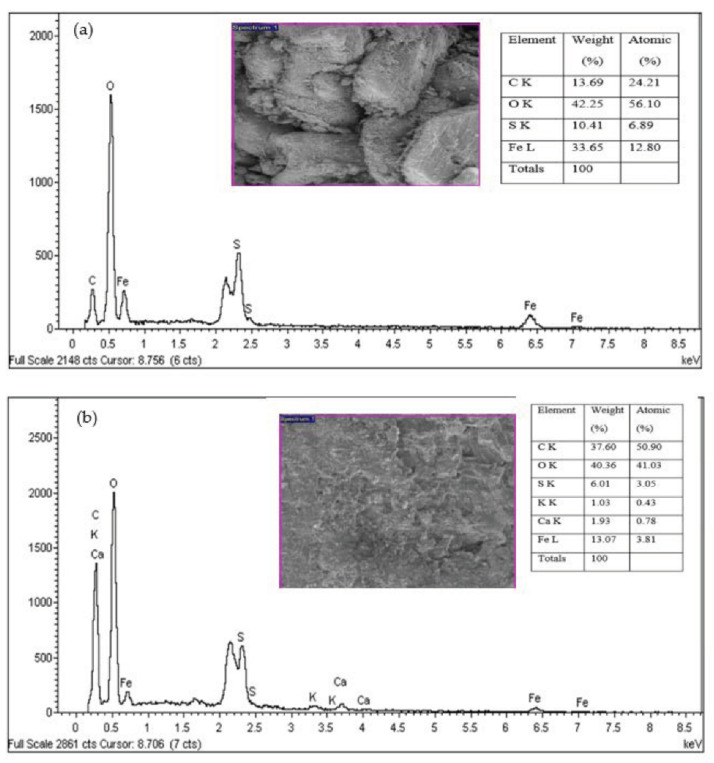
(**a**) SEM-EDX of FeSO_4_·7H_2_O waste, and (**b**) generated sludge after SPRE treatment at optimized experimental conditions.

The residual NH_3_-N and COD concentrations in treated SPRE (16 ± 2 mg/L and 57 ± 4 mg/L, respectively) were lower than the industrial effluent discharge limits (20 mg/L and 180 mg/L, respectively) recommended by DoE, Malaysia [29]. Thus, the FeSO_4_·7H_2_O waste generated as an industrial byproduct in the titanium oxide manufacturing industry could be utilized as a coagulant in industrial effluent treatment, including rubber processing effluent. Figure 3 shows the SEM image of FeSO_4_·7H_2_O waste and the sludge generated after coagulation–flocculation treatment of SRPE using FeSO_4_·7H_2_O waste as a coagulant. It was found that the FeSO_4_·7H_2_O waste has an irregular shape and a rough surface with holes (Figure 3a). However, the surface sludge generated after treating SRPE is smooth and covered with particles (Figure 3a), revealing the adsorption of suspended and colloidal particles present on the surface of the FeSO_4_·7H_2_O waste. The elemental and mineral composition analyses are shown in Figure 4 and Table 7, respectively. The sludge generated after coagulation SRPE using FeSO_4_·7H_2_O waste as a coagulant contains essential plant nutrients, including iron (Fe), phosphorous (P), potassium (K), magnesium (Mg), calcium (Ca), and boron (B). Although coagulation–flocculation has been viewed as the most effective physicochemical treatment process for treating industrial effluent, the generation of toxic sludge has raised environmental pollution concerns. However, iron is an essential micronutrient for plant growth and metabolism. Generally, iron deficiency is a common nutritional disorder of plant growth. Moreover, iron involves in chloroplast synthesis, and it is an essential element to maintain the plant chloroplast function and structure [30,31]. 

Numerous studies have reported using iron-containing sludge as a fertilizer for plant growth and soil erosion mitigation [32,33,34]. Wang et al. [33] determined the feasibility of steel sludge as an iron fertilizer for soil improvement and corn growth. The study reported that the steel slag could be utilized as a promising source for iron to alleviate crop iron chlorosis in iron-deficient calcareous soil. Wang et al. [34] applied the iron sludge in a subtropical paddy field to enhance crop yield and minimize greenhouse gases emission. Thus, the FeSO_4_·7H_2_O waste could be utilized as an environmentally friendly coagulant to treat industrial effluent, including rubber processing effluent. The generated sludge, after coagulation of industrial effluent, would be consumed as a fertilizer in plantations, which will promote zero-waste discharge from treating industrial effluent. 

## 4. Conclusions

In the present study, FeSO_4_·7H_2_O waste from the titanium oxide manufacturing industry was employed as a coagulant for the NH_3_-N and COD removal from SRPE. It was found that the second-order polynomial equation adequately fitted experimental data. The coagulation time, FeSO_4_·7H_2_O waste doses, and temperature were statistically significant for the COD removal from SRPE, while FeSO_4_·7H_2_O waste doses and temperature were statistically significant for the NH_3-_N removal from SRPE. The optimal experimental conditions were determined to be the coagulation time of 70 min, doses 900 mg/L, and the temperature 62 °C. The maximum COD and NH_3_-N removal obtained were 93.86% and 98.19%, respectively, at the optimized FeSO_4_·7H_2_O coagulation experimental conditions. The residual COD and NH_3_-N in treated SPRE were 57 ± 4 mg/L and 16 ± 2 mg/L, respectively, below the stipulated industrial effluent discharge limits set by DoE, Malaysia. Hence, the optimized conditions found in the present study show a satisfactory outcome and can be applied to remove COD and NH_3_-N in treating SRPE generated by the rubber processing industry. The sludge generated after coagulation SRPE contains essential plant nutrients, including iron (Fe), phosphorous (P), potassium (K), calcium (Ca), magnesium (Mg), and boron (B). Thus, the sludge generated from the SRPE treatment using FeSO_4_·7H_2_O waste as a coagulant could be utilized as an organic fertilizer for plant growth. Therefore, it can be postulated that using FeSO_4_·7H_2_O waste as a coagulant would enhance the sustainable utilization of a waste product and promote zero waste generation from industrial effluent treatment. 

## Figures and Tables

**Table 1:** Physicochemical properties of untreated SPRE.

Parameters	Unit	Concentration
pH	-	7.31 ± 0.23
Temperature	°C	28 ± 1
BOD	mg/L	220 ± 5
COD	mg/L	930 ± 12
SS	mg/L	1148 ± 24
NH_3_-N	mg/L	445 ± 6

**Table 2:** Experimental design for the optimization of process variables.

Factor	Symbol	Low (−1)	Intermediate (0)	High (+1)
Time (min)	x_1_	30	45	60
Doses (mg/L)	x_2_	500	750	1000
Temperature (°C)	x_3_	40	50	60

**Table 3:** Actual and predicted values for eliminating COD and NH_3_-N from SRPE using FeSO_4_·7H_2_O waste as a coagulant.

Run	X_1_	X_2_	X_3_	COD Removal (%)		NH_3_-N Removal (%)	
				Actual Value	Predicted Value	Actual Value	Predicted Value
1	−1	−1	−1	74.62	75.90	73.26	76.10
2	+1	−1	−1	81.18	80.40	65.62	66.91
3	−1	+1	−1	83.23	82.29	72.81	70.05
4	+1	+1	−1	86.67	86.95	87.42	87.42
5	−1	−1	+1	80.00	79.69	85.39	86.25
6	+1	−1	+1	81.18	82.10	65.62	70.91
7	−1	+1	+1	87.20	87.96	80.67	81.90
8	+1	+1	+1	91.83	90.53	95.10	94.78
9	−1.68	0	0	84.73	84.25	86.74	86.66
10	+1.68	0	0	89.68	90.20	93.26	89.77
11	0	−1.68	0	71.72	71.05	67.42	62.52
12	0	+1.68	0	82.80	83.51	76.18	77.51
13	0	0	−1.68	82.80	82.89	76.18	75.60
14	0	0	+1.68	89.14	89.09	91.91	88.93
15	0	0	0	87.96	87.97	91.24	91.15
16	0	0	0	87.42	87.97	89.66	91.15
17	0	0	0	89.79	87.97	89.66	91.15
18	0	0	0	87.63	87.97	91.91	91.15
19	0	0	0	87.85	87.97	93.03	91.15
20	0	0	0	87.20	87.97	90.79	91.15

**Table 4:** Regression Coefficient from Linear and Quadratic Model for the Removal of COD and NH_3_-N from SRPE using FeSO_4_·7H_2_O waste as a coagulant.

Term	Coefficient		Standard Error		F-Value		*p*-Value	
	COD	NH_3_-N	COD	NH_3_-N	COD	NH_3_-N	COD	NH_3_-N
Model	87.97	89.49	0.45	1.70	44.40	10.55	<0.0001	0.0001
$x_{1}$	1.77	0.92	0.30	1.13	34.90	0.67	0.0001	0.4336
$x_{2}$	3.70	4.46	0.30	1.13	153.33	15.53	0.0001	0.0001
$x_{3}$	1.84	3.96	0.30	1.13	37.97	12.29	0.0001	0.0001
$x_{1}{}^{2}$	−0.27	−0.47	0.29	1.10	0.83	0.18	0.3838	0.6786
$x_{2}{}^{2}$	−3.78	−6.90	0.29	1.10	168.63	39.37	0.0001	0.0001
$x_{3}{}^{2}$	−0.70	−2.57	0.29	1.10	5.81	5.48	0.0367	0.0413
$x_{1}x_{2}$	0.041	7.06	0.39	1.48	0.11	22.83	0.0180	0.0007
$x_{1}x_{3}$	−0.51	−1.54	0.39	1.48	1.80	1.09	0.2099	0.3220
$x_{2}x_{3}$	0.47	0.43	0.39	1.48	1.44	0.083	0.2580	0.7788

**Table 5:** Analysis of the variance (ANOVA) for removing COD and NH3-N from SPRE using FeSO_4_·7H_2_O waste as a coagulant.

Source	Degree of Freedom	Sum of Squares		Mean Square		F-Value		*p*-Value	
		COD ^1^	NH_3_-N ^2^	COD	NH_3_-N	COD	NH_3_-N	COD	NH_3_-N
Model	9	488.27	1657.02	54.25	184.11	44.40	10.55	0.0001	0.0001
Residual error	10	12.22	174.51	1.22	17.45				
Lack-of-fit	5	7.88	99.78	1.58	19.96	1.82	1.34	0.2640	0.3794
Pure error	5	4.34	74.73	0.87	14.95				
Total	19	500.49	1831.53						

^1^*R^2^* = 0.9756; *R^2^ (adj)* = 0.9536; ^2^ *R^2^* = 0.9047; *R^2^ (adj)* = 0.8190.

**Table 6:** Optimized experimental condition for removing COD and NH_3_-N from SPRE using FeSO_4_·7H_2_O as a coagulant.

Variables	Optimized Experimental Condition	COD Removal (%)		NH_3_-N Removal (%)	
		Predicted	Actual	Predicted	Actual
Time	70 min	94.68	93.86	98.58	98.19
Doses	900 mg/L				
Temperature	62 °C				

**Table 7:** Mineral compositions analyses in sludge generated after coagulation using FeSO_4_·7H_2_O waste as a coagulant.

Minerals	Unit	Amount
Boron (B)	mg/kg	0.14 ± 0.02
Calcium (Ca)	mgkg	2.60 ± 0.45
Iron (Fe)	mg/kg	134.32 ± 1.53
Magnesium (Mg)	mg/kg	18.52 ± 1.78
Phosphorus (P)	mg/kg	14.03 ± 1.71
Potassium (K)	mg/kg	220.63 ± 5.15

## Data Availability

Not applicable.
